# Surgical clipping of a dissecting aneurysm of the precommunicating segment of the anterior cerebral artery: a case report and review of the literature

**DOI:** 10.1186/s13256-015-0604-x

**Published:** 2015-05-23

**Authors:** Oreste de Divitiis, Alberto Di Somma, Teresa Somma, Luigi Maria Cavallo, Mariano Marseglia, Francesco Briganti, Paolo Cappabianca

**Affiliations:** 1 Department of Neurosciences, Reproductive and Odontostomatological Sciences, Division of Neurosurgery, Università degli Studi di Napoli “Federico II”, Via S. Pansini, 5, 80131 Naples, Italy; 2 Department of Advanced Biomedical Sciences, Unit of Interventional Neuroradiology, Università degli Studi di Napoli “Federico II”, Naples, Italy

**Keywords:** Anterior cerebral artery, Cerebral aneurysm, Dissecting aneurysm, Pterional approach, Surgical clipping

## Abstract

**Introduction:**

Dissecting aneurysms of the cerebral arteries are uncommon vascular malformations. Neurosurgical treatment remains critical in the management of patients with such vascular pathologies.

**Case presentation:**

A 20-year-old Caucasian woman presented with a sudden onset of severe headache and loss of consciousness. Computed tomography revealed diffuse subarachnoid hemorrhage, while a computed tomography disclosed a dissecting aneurysm of the precommunicating segment of the right anterior cerebral artery. Cerebral carotid angiography confirmed the presence of the dissecting aneurysm. Due to the peculiar anatomic configuration, endovascular treatment was excluded and surgery was selected. As the left circulation perfused both postcommunicating segments of the anterior cerebral artery and the distal right precommunicating segment was hypoplastic, direct clipping of the right precommunicating segment, close to its origin from the internal carotid artery, was carried out. She recovered after surgery and a late angiography showed the correct positioning of the clip, with regular perfusion of both right and left postcommunicating segments.

**Conclusions:**

The management of dissecting aneurysms of the cerebral arteries is still controversial. With this report we highlight a possible neurosurgical option among therapeutic strategies for these uncommon vascular lesions.

## Introduction

Dissecting aneurysms of the cerebral arteries are infrequent pathological conditions. Most dissecting aneurysms occur in the extracranial vessels and are most commonly due to traumatic events [1].

Nowadays, despite their relatively rare occurrence, intracranial aneurysmal dissections are more frequently diagnosed due to better awareness and increased availability of modern imaging techniques; particular attention has been paid to their pathogenesis, natural history, and optimal management [2]. The etiology and pathogenesis of most dissections involving intracranial vessels are still unclear.

Regarding the specific origin site, dissecting aneurysms of the cerebral arteries tend to occur most commonly in the internal carotid artery (ICA) [3], the middle cerebral artery [4], and the vertebrobasilar system [5,6]. The anterior cerebral artery (ACA) is usually involved in association with dissection in other locations, such as in an ICA dissecting aneurysm, but a lesion confined to the ACA, especially in the precommunicating segment, is an extremely rare event (Table 1) [1,7-18].

**Table 1:** **Clinical characteristics of 13 patients with dissecting aneurysms involving the precommunicating segment of the anterior cerebral artery**

**Authors and Reference number**	**Age (years)/Sex**	**Dissecting aneurysm location**	**Clinical evidence**	**Treatment**	**Complications**	**Outcome**
Gherardi and Lee [7]	26/F	NA	Subarachnoid hemorrhage, headache, coma	NA	–	Death
Nelson [10]	5/M	NA	Headache, right hemiparesis, aphasia	NA	–	Death
Pilz [16]	22/F	NA	Incidental	NA	–	Death
Yamashita *et al*. [1]	16/F	Right A_1_ segment	Confusion, left hemiparesis, left homonymous hemianopsia	Medical therapy (dexamethasone and tranexamic acid)	–	Death
Honda *et al*. [18]	48/F	NA	Headache, right hemiparesis	NA	NA	Good recovery
Hirao *et al*. [17]	58/F	Left A_1_ segment	Headache, loss of consciousness, aphasia, involuntary movements	Trapping and clipping	Low perfusion area of the medial and inferior part of the left frontal lobe	Good recovery
	39/F	Left A_1_ segment	Headache, aphasia, right hemiparesis and facial nerve paresis, confusion	Conservative treatment	NA	Good recovery
Leach *et al*. [13]	39/F	Right A_1_ segment	Confusion, loss of consciousness	Surgical trapping with two straight clips	Ischemia of the right caudate nucleus head	Good recovery
Hasegawa *et al*. [8]	23/M	Right A_1_ segment	Headache	Trapping and resection of the aneurysm	NA	Good recovery
Iwashita *et al*. [12]	53/F	Right A_1_ segment	Left hemiparesis and alien hand syndrome	Trapping of the proximal and distal site of the aneurysm		
Lv *et al*. [14]	43/M	Left A_1_ segment	Loss of consciousness	Endovascular stenting and, 3 months later, complete endovascular occlusion of the left A_1_ portion of the anterior cerebral artery	Regrowth of the aneurysm	Good recovery
Wu and Chiu [15]	NA	A_1_ segment	Visual field defect	Surgical treatment	NA	Good recovery
de Divitiis *et al*. **(present study)**	28/F	Right A_1_ segment	Headache, loss of consciousness	Surgical clipping of the right anterior cerebral artery	Ischemia of the right caudate nucleus head	Good recovery

*Abbreviations: A*_*1*_, precommunicating segment of the anterior cerebral artery; *F,* female*; M,* male*; NA,* not available.

Regarding the neuroimaging, the ACA aneurysmal dissection is more difficult to identify than the vertebrobasilar because of narrower vessel calibers and more curved features. However, key signs include a double lumen, stenosis and dilatation (“pearl and string sign”), stenosis alone (“string sign”) or occlusion.

Because of the rarity of ACA dissecting aneurysms, there are no standardized treatments described in the pertinent literature. However, conservative, endovascular, and surgical approaches can be taken into account as possible strategies for the management of those rare vascular pathologies.

We here report a case of dissecting aneurysm of the A_1_ segment of the ACA which was treated via a direct surgical clipping of the homolateral ACA.

## Case presentation

A 20-year-old previously healthy Caucasian woman was admitted to a local hospital because of the sudden onset of severe headache and loss of consciousness. Computed tomography (CT) revealed diffuse subarachnoid hemorrhage (SAH) involving the basal cisterns and the anterior part of the interhemispheric fissure. The SAH was classified as group 3 according to the Fisher’s scale [19]. She was then referred to our hospital and, upon admission, a neurological examination showed severe headache and nuchal rigidity (Grade II of the Hunt and Hess scale).

**Figure 1:**
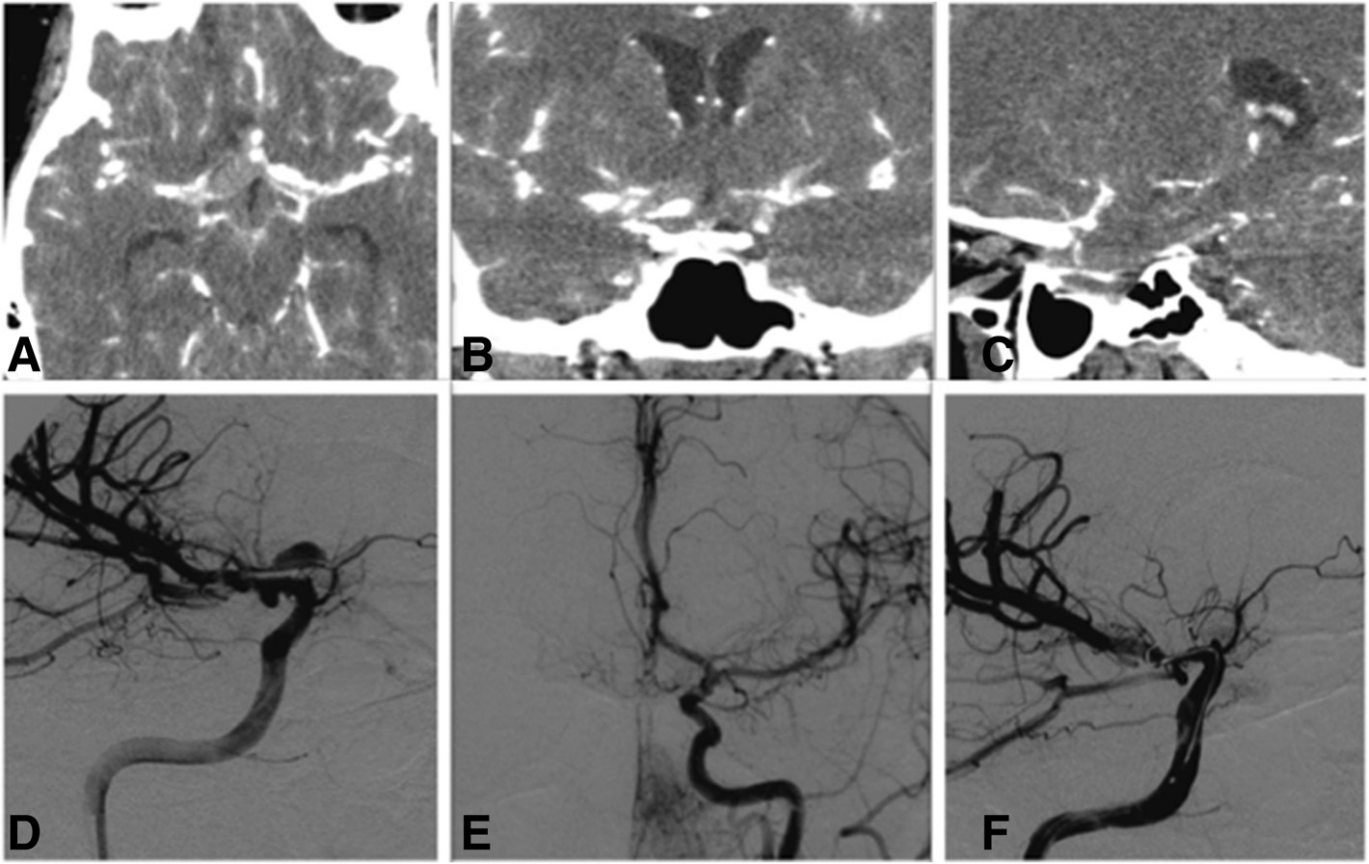
Axial **(A)**, coronal **(B)** and sagittal **(C)** computed tomography angiography scans showing a dilated precommunicating segment of the right anterior cerebral artery. An angiogram of the right internal carotid artery **(D)** showing a false lumen at the level of the precommunicating segment of the right anterior cerebral artery suspected to be a dissecting aneurysm. An angiogram of the left internal carotid artery **(E)** demonstrating that both right and left postcommunicating segments of the anterior cerebral artery are perfused from the left anterior cerebral artery. An angiogram of the right internal carotid artery **(F)** showing the impossibility of accessing the aneurysm via an endovascular route due to its characteristic features and the vasospasm.

Neuroradiological investigation by means of computed tomography angiography (CTA) disclosed a dissecting aneurysm of the A_1_ segment of the right ACA (Figure 1A-C). Digital subtraction angiography confirmed the presence of the dissecting aneurysm of the A_1_ segment of the right ACA originating from the parent vessel with a very acute angle (Figure 1D). No perforating arteries were clearly detected. Moreover, hypoplasia of the distal part of the right A_1_ segment (that is, close to the anterior communicating artery) was highlighted. Both right and left postcommunicating segments (A_2_) were perfused from the left ACA, and a balloon occlusion test of the right ICA was performed in order to validate this condition (Figure 1E).

**Figure 2:**
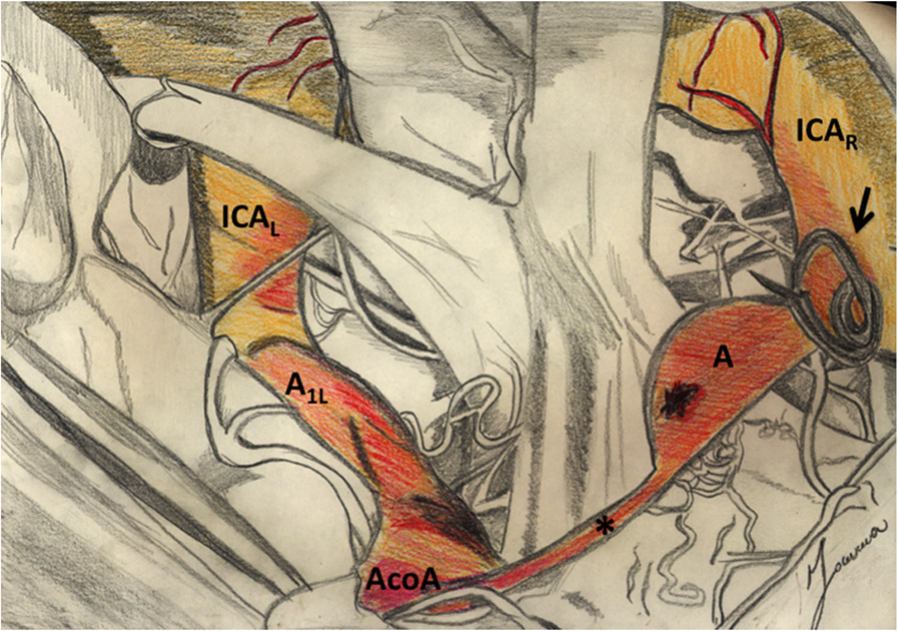
Artist’s drawing describing the surgical clipping of the right precommunicating segment dissecting aneurysm. Abbreviations: A, dissecting aneurysm; A_1L_, precommunicating segment of the left anterior cerebral artery; AcoA, anterior communicating artery; ICA_L_, left internal carotid artery; ICA_R_, right internal carotid artery; *, hypoplasia of the distal part of the precommunicating segment of the right anterior cerebral artery; arrow, surgical clip at the origin of the right precommunicating segment tract.

Because of the characteristic angulation of the aneurysm and the non-accessibility from the opposite site through the anterior communicating artery – due to the hypoplasia of the distal part of the A_1_ segment of the right ACA and to the vasospasm – it was not possible to perform an endovascular treatment (Figure 1F). Accordingly, surgery was chosen by means of clipping the right ACA through a right standard pterional craniotomy (Figure 2) [20]. Using a microsurgical technique the dura mater was opened and reflected anteriorly. Afterwards, with sharp arachnoid dissection the sylvian fissure was opened in a distal-to-proximal direction in order to achieve cerebrospinal fluid release and brain relaxation; these maneuvers allowed us to reduce brain retraction and to visualize the right ICA, with its bifurcation, and the homolateral optic nerve. Finally, a vascular clip was positioned at the origin of the right ACA.

Postoperatively, her headache progressively diminished and left-side weakness initially presented (Grade 3 of the Medical Research Council scale); her left-side weakness was relieved by medical therapy with dihydropyridine calcium channel blocker (nimodipine) and corticosteroids. No other medications were used.

**Figure 3:**
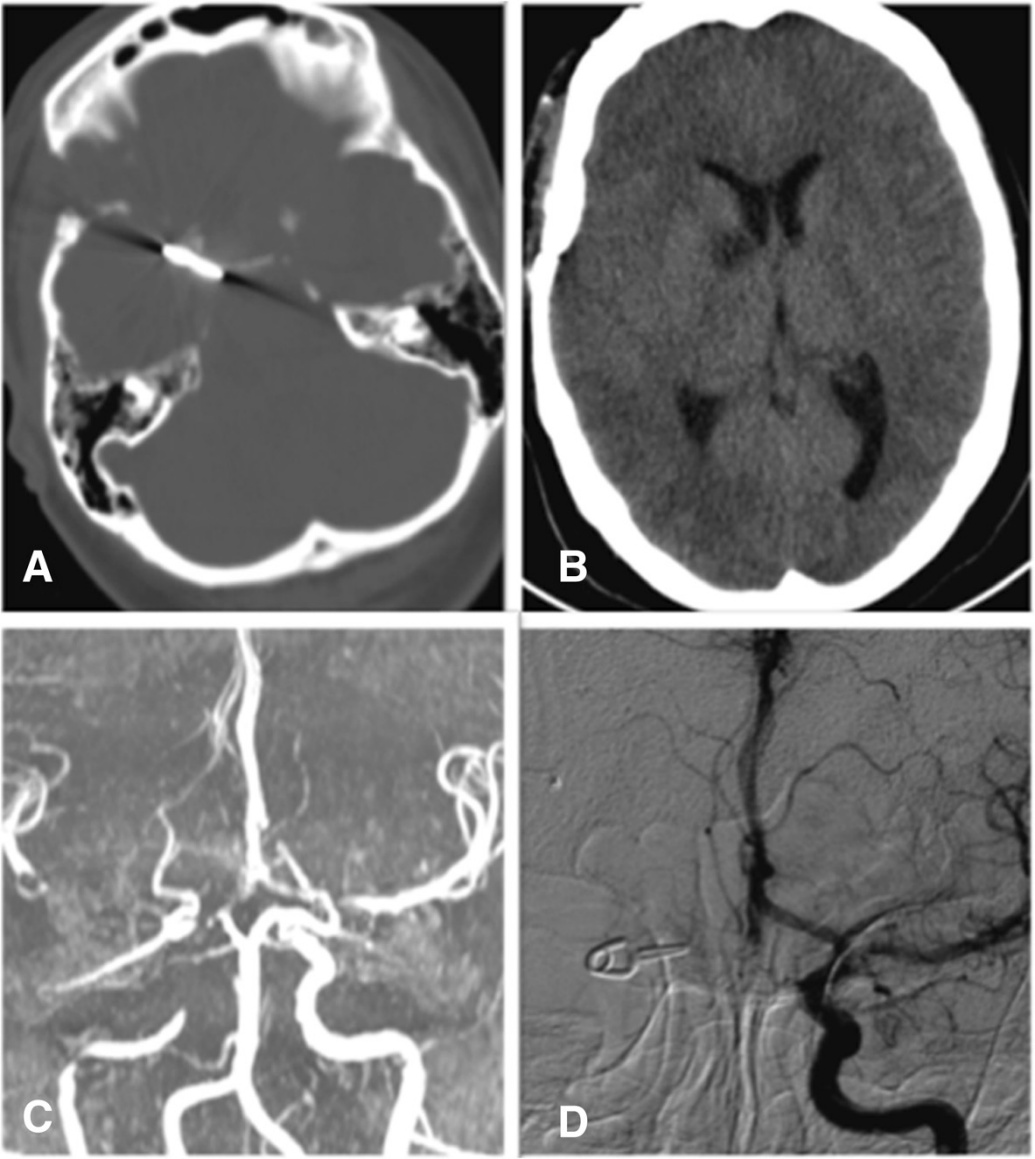
Computed tomography scans postoperative day 3 **(A)** showing the presence of the surgical clip at the level of the right anterior cerebral artery. Computed tomography scans postoperative day 7 **(B)** demonstrating right frontobasal hypodensity area – as per subacute ischemic stroke – and progressive resorption of the subarachnoid hemorrhage. Magnetic resonance angiography 1-month follow up **(C)** showing the regular perfusion of the areas normally supplied by the Circle of Willis. Three-months angiogram **(D)** highlighting the correct positioning of the clip, with regular perfusion of both right and left anterior postcommunicating cerebral arteries by the left carotid circulation.

CT scans, performed at postoperative days (PODs) 3 and 7, showed a right frontobasal hypodensity area – as per subacute ischemic stroke – and progressive resorption of the SAH (Figure 3A-B). Early postoperative CTA- magnetic resonance imaging scans (1 month) confirmed and characterized the right frontobasal subacute ischemia with regular flow of the anterior cerebral circulation (Figure 3C).

Neurologic examination remained otherwise unchanged and she was discharged on POD 24 without any new neurological defect.

A late postoperative angiography (3 months) showed the correct positioning of the clip, with regular perfusion of both right and left anterior postcommunicating cerebral arteries (Figure 3D).

Six months after surgical treatment she showed no clinical and/or neurological defects of new onset and resumed her ordinary life.

## Discussion

The appropriate management of anterior circulation dissecting aneurysms remains controversial. Conservative treatment could be effective with a good outcome and a low rate of second rupture but, if there is a high risk of rebleeding (growing dissecting aneurysm, giant dissecting aneurysm or dissection associated with uncontrolled hypertension), and/or severe clinical conditions arise, direct treatment of the dissecting aneurysm should be proposed.

Nowadays, multimodal treatment for complex cerebral aneurysms includes two major options: endovascular procedures (that is, coiling, stent-assisted coiling, and flow diversion stents) and direct neurosurgical approach (that is, clipping with or without extra-intracranial bypass).

During the last decade, the management of ruptured and unruptured intracranial aneurysms is moving from neurosurgical clipping to endovascular embolization as the preferred, safe and effective treatment modality.

In our case, endovascular access was unfeasible due to the extremely small size of the parent vessel and the acute angle of origin of the aneurysm. Furthermore, the particular anatomical condition of the right A_2_, perfused by the opposite side, determined the choice of a direct surgical approach by means of a right pterional craniotomy and clipping at the origin of the right A_1_ tract. It should be also stressed that other surgical options can be considered. For example, wrapping, that is, wrap the aneurysm with materials (muscle, Teflon®) to promote scarring or trapping, that is, both distal and proximal arterial interruption with direct surgery (ligation or occlusion with a clip) or bypass surgery were not considered. Indeed, in the present case, the left anterior circulation perfused both A_2_ segments and the distal part of the right A_1_ segment was hypoplastic.

This paper is intended to highlight that vascular neurosurgeons and interventional radiologists must consider a multitude of factors when developing the best treatment option for an individual patient. Optimal management requires a thorough understanding of the anatomy and natural history of such aneurysms as well as risks and benefits related to the different treatment modalities.

## Conclusions

The management of cerebral dissecting aneurysms is still controversial. With this report we highlight a possible neurosurgical option among therapeutic strategies for these uncommon vascular lesions.

## Consent

Written informed consent was obtained from the patient for publication of this case report and any accompanying images. A copy of the written consent is available for review by the Editor-in-Chief of this journal.
